# Direct Detection of *penA* Gene Associated with Ceftriaxone-Resistant *Neisseria gonorrhoeae* FC428 Strain by Using PCR

**DOI:** 10.3201/eid2408.180295

**Published:** 2018-08

**Authors:** David M. Whiley, Lebogang Mhango, Amy V. Jennison, Graeme Nimmo, Monica M. Lahra

**Affiliations:** The University of Queensland, Brisbane, Queensland, Australia (D.M. Whiley, L. Mhango);; Pathology Queensland Central Laboratory, Brisbane (D.M. Whiley, G. Nimmo);; Queensland Health Forensic and Scientific Services, Archerfield, Queensland, Australia (A.V. Jennison);; Griffith University, Gold Coast, Queensland, Australia (G. Nimmo);; The Prince of Wales Hospital Randwich, Sydney, New South Wales, Australia (M.M Lahra);; The University of New South Wales, Sydney (M.M. Lahra)

**Keywords:** Neisseria gonorrhoeae, FC428 clone, penA, penicillin-binding protein 2, PBP2, ceftriaxone, antimicrobial resistance, oligonucleotide, *N*. *lactamica*, *N*. *meningitides*, Japan, Denmark, Canada, Australia

## Abstract

The ceftriaxone-resistant *Neisseria gonorrhoeae* FC428 clone was first observed in Japan in 2015, and in 2017, it was documented in Denmark, Canada, and Australia. Here, we describe a PCR for direct detection of the *penA* gene associated with this strain that can be used to enhance surveillance activities.

Ceftriaxone, either monotherapy or in dual therapy with azithromycin, is the mainstay of treatment of patients diagnosed with *Neisseria gonorrhoeae* infection in most settings (*1*). Therefore, the identification of any strains exhibiting resistance to ceftriaxone is of considerable public health concern. Until 2017, ceftriaxone-resistant strains of *N. gonorrhoeae* had been rare and typically sporadic, including in 2009, H041 in Japan (*2*); in 2010, F89 in France (*3*); in 2011, F89 in Spain (*4*); in 2013, A8806 in Australia (*5*); in 2014, GU140106 in Japan (*6*); and in 2015, FC428 and FC460 in Japan (*7*). However, there is now evidence of sustained international transmission of FC428, reported during 2017 in Canada (*8*) and Demark (*9*) (1 case each) and in Australia (2 cases) (*10*). Rapid and timely detection is pivotal to contain further spread of antimicrobial drug–resistant *N. gonorrhoeae*. Here, we describe a real-time PCR protocol to facilitate enhanced surveillance for the FC428 clone. The study was approved by the University of Queensland Human Research Ethics Committee.

## The Study

We designed a real-time PCR to target unique sequences on the *penA* gene of the FC428 *N. gonorrhoeae* clone (*10*). Modifications of *penA*, which encodes penicillin-binding protein 2 (PBP2), are a cause of cephalosporin resistance in *N. gonorrhoeae*. The FC428 clone harbors a mosaic *penA*-allele, designated as PenA-60.001 by results of *N. gonorrhoeae* sequence typing for antimicrobial drug resistance (*10*), and encodes alterations including A311V and T483S that have previously been associated with *N. gonorrhoeae* ceftriaxone resistance in H041 (*2*) and A8806 (*2*,*5*) strains. For this study, we designed 2 primers and 2 allele-specific probes (Table 1) to facilitate specific detection of the *penA* gene of FC428. In brief, the forward and reverse primers were designed to flank the A311V alteration; probe 1 was designed for detection of the A311V alteration; and probe 2 was designed to detect the wild-type A311 sequence. We added probe 2 to act as a blocker probe to limit binding of probe 1 with the wild-type sequence.

**Table 1:** Primer and probe sequences for PCR to detect *Neisseria gonorrhoeae* FC428 strain*

Designation	Oligonucleotide sequence, 5′ → 3′
Forward primer	CGCAACCGTGCCGTT
Reverse primer	GGGTATTGAATGTGTCTGTTGGA
Probe 1	Fam-TTCA+T+G+A+CA+G+AAC-Iowa Black FQ
Probe 2	Hex-TCA+T+G+G+CA+GA-Iowa Black FQ

*LNA bases are indicated by + preceding the base in the sequence.

We prepared the reaction mix by using the QuantiTect Probe PCR Master Mix Kit (QIAGEN, Doncaster, Victoria, Australia). The reaction consisted of 12.5 µL of the Master Mix, 10 pmol/L of forward and reverse primers (Table 1), 4.0 pmol/L of each probe, and 5.0 µL of specimen nucleic acid, resulting in a total volume of 25 µL. We thermocycled the reaction mix by using the Rotor-Gene 6000 instrument (QIAGEN) and held it at 95°C for 15 min, then cycled (45 cycles) at 95°C for 15 s and 60°C for 60 s. We analyzed data by using the Rotor-Gene allelic discrimination software (QIAGEN).

We initially assessed the analytical performance of the assay by testing cultured isolates of *N. gonorrhoeae* (n = 72) and commensal *Neisseria* and *Moraxella* species (n = 111) (Table 2). We prepared these isolates by using a previously described heat-denaturation method (*11*). The *N. gonorrhoeae* isolates included the 2 FC428 strains recently documented in Australia (*10*), H041 (*2*) and A8806 (*5*); the ceftriaxone-resistant strains; and other local clinical *N. gonorrhoeae* isolates (n = 68). Both FC428 isolates provided strong positive signals by using probe 1 with cycle threshold (C_t_) values <20 cycles. The A8806 strain provided a late reaction at 37.8 cycles (probe 1), as did 2 commensal *Neisseria* strains: 1 *N. lactamica* isolate at 42.8 cycles for (probe 1), and 1 *N. meningitidis* isolate at 32.6 cycles (probe 2). The Figure shows a sequence alignment of the partial *penA* sequences from these 3 isolates compared with the FC428 PenA-60.001 allele. The A8806 strain shows considerable sequence homology with PenA-60.001 (including 100% match with the A311V Probe 1 sequence), albeit for 2 mutations in the forward primer designed to limit detection of A8806. We do not consider this a limitation of the assay because there has only been 1 reported case of infection with the A8806 strain. Neither the *N. lactamica* or *N. meningitidis* isolates harbored the A311V alteration.

**Table 2:** *Neisseria* spp. isolates and specimens tested in development of PCR to detect *Neisseria gonorrhoeae* FC428 strain*

Isolates/samples	PCR results for FC428 (C_t_)	
Probe 1	Probe 2
Gonococcal species, n = 144		
*Neisseria gonorrhoeae*, n = 72		
*N. gonorrhoeae* FC428, n = 2 (*9*)†	Positive (19.8 and 18.17 cycles)	Negative
*N. gonorrhoeae* H041, n = 1 (*1*)	Negative	Negative
*N. gonorrhoeae* A8806, n = 1 (*4*)	Positive (37. 8 cycles)	Negative
*N. gonorrhoeae*, n = 68‡	Negative	Negative
Nongonococcal species, n = 111		
*N. cinerea*, n = 4	Negative	Negative
*N. elongata*, n = 1	Negative	Negative
*N. flavescens,* n = 1	Negative	Negative
*N. lactamica,* n = 15	Negative	Negative
*N. lactamica,* n = 1	Positive (42.8 cycles)	Negative
*N. meningitidis,* n = 55	Negative	Negative
*N. meningitidis,* n = 1	Negative	Positive (32.6 cycles)
*N. mucosa,* n = 1	Negative	Negative
*N. polysacchareae*, n = 4	Negative	Negative
*N. sicca,* n = 4	Negative	Negative
*N. subflava,* n = 14	Negative	Negative
*N. weaveri*, n = 1	Negative	Negative
*Moraxella catarrhalis*, n = 7	Negative	Negative
*M. osloensis*, n = 2	Negative	Negative
*N. gonorrhoeae* NAAT-positive clinical specimens, n = 358		
Urogenital, n = 172	Negative	Negative
Anal swab, n = 81	Negative	Negative
Throat swab, n = 95	Negative	Negative
Other, n = 10	Negative	Negative

*Ct, cycle threshold; NAAT, nucleic acid amplification test. †Isolates A7846 and A7536. ‡Other local clinical isolates collected in New South Wales, Australia.

**Figure:**
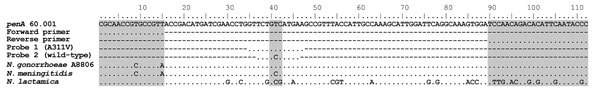
Sequence alignment showing the expected 112-bp PCR product for the PCR to detect *Neisseria gonorrhoeae* FC428 strain. PenA type 60.001 is provided as the reference sequence. Gray indicates the primer targets and the 311 codon within the probe target sequences. The *penA* sequences from the *N. gonorrhoeae* A8806, *N. meningitidis*, and *N. lactamica* isolates that cross-reacted with the FC428 PCR are also provided. Dots indicate sequence identity.

To compare detection limits, we tested 10-fold dilutions of FC428 DNA by both the FC428 PCR and a previously described in-house *N. gonorrhoeae* PCR, directed at the gonococcal *porA* and *opa* sequences (*12*). The in-house *N. gonorrhoeae* PCR had the lowest detection limit at 0.3 genome copies/reaction, whereas the detection limit of the FC428 PCR was 3.0 genome copies/reaction, indicating the FC428 PCR was 1 log less sensitive than the diagnostic method.

We then applied the assay to a convenience panel of *N. gonorrhoeae*–positive clinical samples (n = 358) submitted to Pathology Queensland Laboratory (Brisbane, Queensland, Australia) during February–September 2017 (Table 2). In brief, these samples comprised remnant nucleic acids from samples that tested positive for *N. gonorrhoeae* by the Cobas 4800 CT/NG test and were confirmed positive by using the in-house *N. gonorrhoeae* PCR (*12*). All samples provided negative results by the FC428 PCR, suggesting that the FC428 strain was not present in Queensland during this period.

## Conclusions

Overall, our results suggest that the FC428 PCR is suitable for screening for the FC428 *N. gonorrhoeae* clone in clinical specimens for which culture is not available. The method could prove to be a strategic tool to enhance surveillance if FC428 continues to spread. We recommend that positive results be confirmed by, for example, DNA sequencing, particularly if the strain is detected in a pharyngeal sample in which other commensal *Neisseria* species are prevalent.
